# Getting into a “Flow” state: a systematic review of flow experience in neurological diseases

**DOI:** 10.1186/s12984-021-00864-w

**Published:** 2021-04-20

**Authors:** Beatrice Ottiger, Erwin Van Wegen, Katja Keller, Tobias Nef, Thomas Nyffeler, Gert Kwakkel, Tim Vanbellingen

**Affiliations:** 1 Neurocenter, Luzerner Kantonsspital, Spitalstrasse 31, 6000 Luzern 16, Switzerland; 2 Department of Rehabilitation Medicine, Amsterdam Movement Sciences, Amsterdam UMC, VU University Medical Center, Amsterdam, The Netherlands; 3 Amsterdam Neuroscience, Vrije Universiteit, Amsterdam, The Netherlands; 4 ARTORG Center for Biomedical Engineering Research, Gerontechnology and Rehabilitation Group, University Bern, 3008 Bern, Switzerland; 5 Department of Physical Therapy and Human Movement Sciences, Northwestern University, Evanston, IL USA

**Keywords:** Systematic review, Flow experience, Gaming, Neurological diseases

## Abstract

**Background:**

Flow is a subjective psychological state that people report when they are fully involved in an activity to the point of forgetting time and their surrounding except the activity itself. Being in flow during physical/cognitive rehabilitation may have a considerable impact on functional outcome, especially when patients with neurological diseases engage in exercises using robotics, virtual/augmented reality, or serious games on tablets/computer. When developing new therapy games, measuring flow experience can indicate whether the game motivates one to train. The purpose of this study was to identify and systematically review current literature on flow experience assessed in patients with stroke, traumatic brain injury, multiple sclerosis and Parkinson’s disease. Additionally, we critically appraised, compared and summarized the measurement properties of self-reported flow questionnaires used in neurorehabilitation setting.

**Design:**

A systematic review using PRISMA and COSMIN guidelines.

**Methods:**

MEDLINE Ovid, EMBASE Ovid, CINAHL EBSCO, SCOPUS were searched. Inclusion criteria were (1) peer-reviewed studies that (2) focused on the investigation of flow experience in (3) patients with neurological diseases (i.e., stroke, traumatic brain injury, multiple sclerosis and/or Parkinson’s disease). A qualitative data synthesis was performed to present the measurement properties of the used flow questionnaires.

**Results:**

Ten studies out of 911 records met the inclusion criteria. Seven studies measured flow in the context of serious games in patients with stroke, traumatic brain injury, multiple sclerosis and Parkinson’s disease. Three studies assessed flow in other activities than gaming (song-writing intervention and activities of daily living). Six different flow questionnaires were used, all of which were originally validated in healthy people. None of the studies presented psychometric data in their respective research population.

**Conclusion:**

The present review indicates that flow experience is increasingly measured in the physical/cognitive rehabilitation setting in patients with neurological diseases. However, psychometric properties of used flow questionnaires are lacking. For exergame developers working in the field of physical/cognitive rehabilitation in patients with neurological diseases, a valid flow questionnaire can help to further optimize the content of the games so that optimal engagement can occur during the gameplay. Whether flow experiences can ultimately have positive effects on physical/cognitive parameters needs further study.

**Supplementary Information:**

The online version contains supplementary material available at 10.1186/s12984-021-00864-w.

## Background

Flow experience is a subjective psychological state that people report when they are completely involved in something to the point of forgetting time and their surrounding except the activity itself [1, 2]. During flow, subjective perception of time may change: Time can pass faster or slower and the environment is hardly or no longer perceived. Attention is fully invested in the task at hand, and the person functions at his or her fullest capacity. The flow state was first described by Csikszentmihalyi (1975) as the “optimal experience”. He began his research on flow experiences with the simple question of why people are often highly committed to activities without obvious external rewards. Csikszentmihalyi’s first studies involved interviews with people from different backgrounds such as athletes, chess masters, rock climbers, dancers, composers of music and many more [3]. Csikszentmihalyi and his colleagues developed the “Flow-theory” with general attributes of an optimal experience and its proximal conditions. The Flow-theory proposes nine key characteristics: challenge-skill balance (balance between the challenge of the activity and personal skills), action-awareness merging (involvement in the task; actions become automatic), clear goals (clear idea of what needs to be accomplished), unambiguous feedback (clear and immediate feedback), concentration on task at hand (complete focused on the task), sense of control (clear feeling of control), loss of self-consciousness (no concerns with appearance, focused only the activity), transformation of time (altered perception of time; either speeding up or down), and autotelic experience (the activity is intrinsically rewarding) [2, 4]. Many researchers tried to adapt the Flow-theory [5] and explored predictors and consequences of flow, but its definition and key characteristics as shortly described above, remained largely the same. In fact, a recent paper about flow clearly advocates Csikszentmihalyi’s Flow-theory as the only valid and default conceptualization so far [5].

Because flow experience is associated with elements such as motivation, peak performance, peak experience and enjoyment, the Flow-theory was further explored in various research fields, such as sports, educational science, work and software engineering for gaming [6–9]. Positive associations were found between athletes’ flow experience and their performance measures, indicating that positive psychological flow states are related to increased levels of performance. In addition, significant prediction of the athletes’ performance could be made based on the level of flow experience during the competition [10].

Attempts to systematically measure flow experience started in the 1990’s. Self-reported flow questionnaires were used to measure flow during specific activities, such as computer interactions among students and accountants [11], and among athletes practicing various sports such as basketball, athletics, hiking, jogging and other types of sports [4]. In the past 30 years, different flow questionnaires were developed [12, 13]. They focussed either on the dispositional or core flow experience (tendency to experience flow in general) [14] or on the state flow experience (flow experience in a specific activity) [4]. This lead to some disagreement in literature about how flow actually should be measured, and as well as to the context and task in which a flow questionnaire should be applied [5].

Interestingly, over the last decade, several computer or tablet-based serious games, and virtual/augmented reality therapeutic training applications have been developed that integrate many of the key flow characteristics mentioned above. Furthermore, various studies evaluated the player’s flow experience with questionnaires when applying these newer technologies [15–17]. Serious games are intentionally programmed so that the goals are presented very clearly (i.e., visually through nice icons), and that the requirements of the exercises are adaptable according to the level of player performance. Also, the exercises should be both exciting and attractive enough to maintain the player’s attention. In this way, the player obtains a certain automatic feeling of flow while having full control over his or her actions. These games are sometimes so well designed that one loses track of time. Serious games, robotics, virtual/augmented reality, have found their way into neurorehabilitation [18–21], and theory of flow experience emerged in recent neurorehabilitation studies [22, 23]. Indeed, serious exergames may have an explicit educational and/or therapeutic purpose and are often designed in such a way that they may also improve cognitive or physical capabilities [22, 24]. Interestingly, exergame developers began to look at new games from the perspective of flow experience in order to adapt the game conditions of the players, and used flow questions to assess the users’ engagement for the new therapy form [23, 25]. To assess flow experience during a therapeutic session with a patient, valid questionnaires are needed which may guide a clinician in adapting the level of difficulty, attractiveness, amount of feedback of an exercise, possibly further attributing to an optimal flow experience. Such optimization of the motor learning environment may enhance therapeutic efficacy during an individual training session.

However, to date, there is no consensus on how flow experience should be measured in neurologically impaired patients. Furthermore, no systematic overview exists so far, about current existing flow questionnaires and their psychometric properties. Therefore, the first aim of the present study was to identify and systematically review current literature on flow experience assessed in patients with acquired neurological diseases such as stroke, traumatic brain injury (TBI), multiple sclerosis (MS) and Parkinson’s disease (PD). The second aim was to critically appraise, compare and summarize the measurement properties of self-reported flow questionnaires used in a neurorehabilitation setting. Since flow experience has been assessed already in neurological rehabilitation and measurement tools exist, we expected these tools to be well validated.

## Methods

This systematic review followed the guideline from the Preferred Reporting Items for Systematic Reviews and Meta-Analyses Statement (PRISMA) [26]. The Consensus-based standards for the selection of health measurement instruments (COSMIN guidelines) were applied for the evaluation of the measurement properties of the flow questionnaires [27]. A flow questionnaire is a research instrument consisting of a series of questions for the purpose of gathering information from respondents about their flow experience when performing an activity.

### Protocol and registration

The protocol was registered with the International prospective register of systematic review (PROSPERO) https://www.crd.york.ac.uk/prospero/display_record.php?ID=CRD42020187510 on July 5, 2020 [28].

### Electronic search strategy

Databases were searched up from date of inception (1975) to June 2020 (MEDLINE Ovid, EMBASE Ovid, CINAHL-EBSCO, SCOPUS). Text words and MeSH (Medical Subject Headings) terms for flow experience, flow questionnaire, flow theory, positive psychology, neurorehabilitation, neurological disease, stroke, traumatic brain injury, multiple sclerosis and Parkinson’s disease to identify intervention studies which used flow as outcome parameter. References of the included studies were screened for additional articles. The search strategy was created by one author (KK) and peer reviewed by another author (BO).

The PubMed search strategy was as follows: (flow exp*) NOT (cereb* flow OR dyn* flow OR exp* flow OR blood flow OR venous flow)) AND (stroke OR Parkinson OR traumatic brain injury OR multiple sclerosis). The search string was adapted appropriately for each database (Additional file 1).

### Eligibility criteria

According to PRISMA guidelines [26], the Population-Intervention-Comparison-Outcome-Study Design (PICOS) approach was applied to systematically define the eligibility criteria. Inclusion and exclusion criteria are presented in Table 1.

**Table 1:** Inclusion and exclusion criteria defined in the PICOS framework

	Description	Inclusion/Exclusion criteria
Population	Patients with neurological disease such as stroke, TBI, MS and/or PD	The study sample or a substantial number of subjects (minimal 50%) are represented in the study population. The patients had to be adult. Studies with children and/or adolescents were excluded
Intervention	Instrumented measurements to assess flow experience in a rehabilitation setting	Studies that measured flow using a questionnaire were included. Other ways of measuring flow, such as the Experience-Sample Method or interviews were not included
Comparison	No control group or comparison is required	Comparison to a clinical test, a control group or the effect of intervention related to flow experience will be reported
Outcome	Outcome measured flow experience	The studies had to assess the construct Flow with reference to Flow-theory by Csikzentmihalyi. Studies that measured intrinsic motivation or any other construct of motivation or positive psychology were excluded
Study design	Peer-reviewed studies are included	No restrictions on the type of studies, including case studies, case–control studies, cohort studies, randomized control studies and non/randomized control studies Articles published in languages other than English were excluded

### Selection of studies

Two reviewers (BO, KK) independently screened all titles and abstracts for the eligibility criteria. The full text papers of relevant studies were obtained if both reviewers agreed for inclusion. Otherwise, a third reviewer (TV) made the final decision. The search results were imported into Mendeley Reference Manager (https://www.mendeley.com) to further check for duplicates. In addition, we obtained the original validation papers of each flow questionnaire. These validation papers were used to critically appraise the validity, reliability, and responsiveness of the flow questionnaires.

## Results

**Fig. 1:**
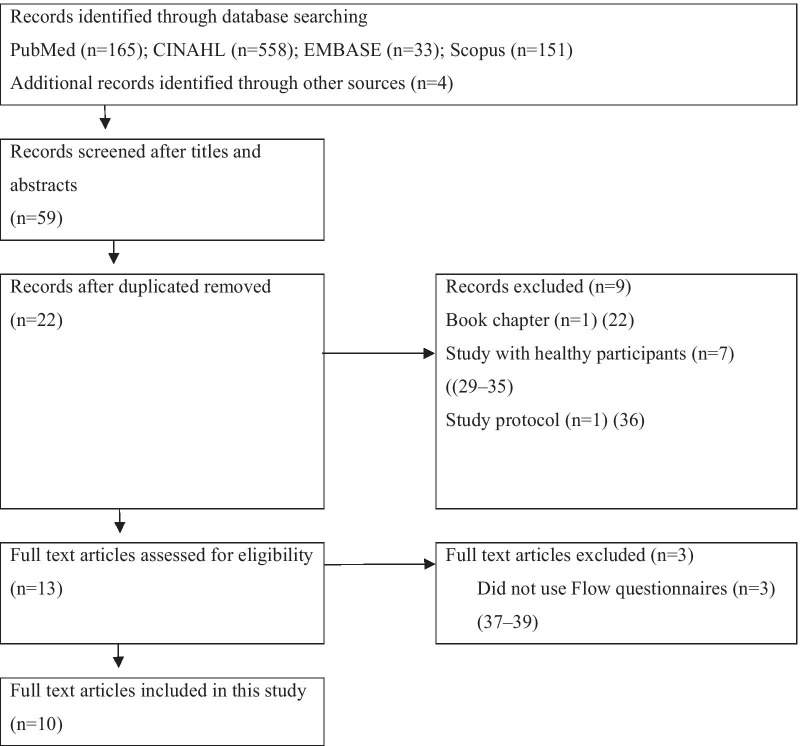
Flow diagram for study selection

The Electronic search strategy identified 911 records, of which 22 were retrieved in full text for further assessment. This resulted in the exclusion of another twelve studies (Fig. 1). Ten studies were included in the review.

### Data extraction and assessment of methodological quality

The general characteristics of the included studies were extracted as following: population (diagnosis, sample size, age, gender), study design, intervention (therapeutic activity in a rehabilitation setting), main outcomes parameters, flow measurement and key findings regarding flow experience. The results are presented in Table 2. The characteristics of the flow questionnaires used, such as the flow construct, mode of administration/instruction, subscales (items) and response option were extracted and are listed in Table 3. Furthermore, we evaluated the measurement properties of the flow questionnaires by assessing the content validity (including relevance, comprehensiveness and comprehensibility of the construct, population and context of use in order to apply the flow questionnaires in a neurorehabilitation setting), construct validity (including structural validity, hypotheses testing, and cross-cultural validity), reliability (containing the measurement properties internal consistency and measurement error and test–retest) and responsiveness (the ability of the flow questionnaires to detect change over time in the flow experience) following the COSMIN guidelines [27]. We verified whether the content of the questionnaires was an adequate reflection of the flow construct. For this purpose, we recorded if the target population was asked about the relevance, comprehensiveness, and comprehensibility of the flow questionnaire (content validity). Regarding construct validity, we examined if the scores of the flow questionnaire were an adequate reflection of the dimensionality of the flow construct (structural validity). We also investigated if the scores of the questionnaires were consistent with hypotheses based on the assumption that the questionnaires validly measure the flow construct (hypotheses testing). Additionally, we investigated if the performance of the items on a translated or culturally adapted questionnaire were an adequate reflection of the performance of the items of the original version of the questionnaire (cross-cultural validity). The domain reliability refers to the degree to which the measurement is free from measurement error. For this reason, we reviewed the degree of the interrelatedness among the items (internal consistency) and the proportion of the total variance in the measurements which was due to true differences between patients (reliability). The results and the psychometric properties’ rating criteria of the flow questionnaires are presented in the Additional file 2. The Summary of Findings (SoF) per measurement property, its overall rating and the grading of the quality of evidence are presented in Table 4. The COMSIN guidelines [27] were applied for the rating of the SoF.

**Table 2:** Characteristics of included studies

References	Sample characteristics												
	Population/N	Study Design/N	Intervention	Age (Years) Mean (SD)/Gender	Main outcome parameters	Flow measurement	Key findings regarding flow						
Shin et al. (2014) [45]	Acute or subacute and chronic stroke	Prospective Cohort Study (N = 20)	Upper limb training with an interactive game-based virtual reality rehabilitation system RehabMaster™ 20 min of RehabMaster™ sessions twice a week for 2 weeks	nr/nr	Upper limb motor function (Fugl-Meyer Assessment (FMA) and modified Barthel Index (MBI)	6 ‘flow’ statements were taken from a 12 item Flow scale [11]	Flow scores improved between different training sessions using the RehabMaster™						
							Flow statements 1. attentional focus 2. attentional focus 3. intrinsic interest or pleasure 4. intrinsic interest or pleasure 5. control 6. control			Mean (SD) 0.8 (1.3) 0.6 (1.1) 0.5 (0.8) 4.3 (1.2) 4.1 (1.0) 0.9 (1.0)		p-value < .01 < .01 < .01 < .01 < .01 < .01	
Galna et al. (2014) [43]	Parkinson’s disease (Hoehn & Yahr stage I–III)	Prospective Cohort Study (N = 9)	Dynamic postural control training with exergame Microsoft Xbox Kinect Duration: 30 min in a Movement Laboratory	68.22 (range 54–78) 6 females 3 males	Semi-structured interview regarding safety and feasibility of the game	Flow State Scale (FSS)	Flow remained at a high-level during gameplay. High scores indicate high level of flow						
							Subscales Autotelic experience (AE) Clear goals (CG) Challenge-skill balance (CB) Concentration on Task (CT) Paradox of Control (PC) Unambiguous Feedback (UF) Action Awareness Merging (AM) Transience of Time (TT) Loss of self-consciousness (LS)			Mean (SD) 4.03 (0.88) 4.22 (0.88) 3.78 (0.96) 4.56 (0.51) 3.44 (0.98) 3.89 (0.87) 3.11 (1.10) 2.67 (1.14) 4.14 (1.06)			
van der Kuil et al. (2018) [42]	Acquired brain injury (Cerebrovascular accident (n = 16) TBI (n = 9) Brain tumor (n = 4) Brain hypoxia (n = 1)	Prospective Cohort Study (N = 30)	Cognitive training using computer-based serious Game (game was constructed in the Unity 3D game engine) Experimental session approximately 60 min of testing	47.2 Range 23 -68 15 females 15 males	Movement control task to assess usability differences between mouse controlled and keyboard controlled Instruction modality between text-based instructions or video-based instructions Feedback timing to assess the effect of cumulative versus delayed feedback on performance and motivation	Overall appreciation questionnaire with six items adapted from the Flow State Scale (FSS) and three items constructed in context of the usability test	Flow scores were high as measured on a Likert scale [1–5]						
							Subscale: Ease of use Enjoyment Clear goals Rewarding Control Attention Concentration Willingness to play again Challenge			Mean (SD) 3.63 (0.25) 4.17 (0.23) 4.00 (0.24) 3.92 (0.22) 3.29 (0.26) 4.79 (0.10) 4.54 (0.19) 4.13 (0.23) 4.08 (0.21)			
Yoshida et al. (2014) [47]	TBI (Patient A 948 days since injury; Patient B 228 days since injury)	Exploratory case study (AB-Design) (N = 2)	Attentional Training with Video game tasks	Patient A: female, 47 years Patient B: male, 41 years	SDMT, TMT-A and B, RAVLT, Continuous Performance Test X task (CPT-X) Moss Attention Rating Scale (MARS)	Flow State Scale for Occupational Tasks (FSSOT)	Flow scores were at high levels and even increased, based on a visual analysis, scores above the mean more than +—SD) after training, and this in both patients						
Baker et al. (2015) [52]	Spinal cord injury and acquired brain injury (in-patients)	Non-rando-mized quasi-experi-mental design (N = 10)	Song writing program Therapists and participant co-created three songs Duration: 12 sessions (twice weekly,1 h) Main outcome: self-concept, various well-being Measures	38.90 (13.21) 1 female 9 males	Head Injury Semantic Differential Scale (HISDS) self-concept Various well-being measures Flourishing Scale, Satisfaction with Life Scale (SWLS), Emotion Regulation Questionnaire (ERQ), Positive Affect and Negative Affect Scale (PANAS-20), Patient Health Questionnaire-9 (PHQ-9), Generalized Anxiety Disorder scale (GAD-7)	Short Flow Scale (SFS) Core Flow Scale (CFS)	Flow scores were high, mean values > 4 points (measured on a Likert scale of 1–5) for the intervention. The scores did not significantly correlate whether with HISDS nor with well-being measures (Flourishing Scale; SWLS; ERQ; PANAS-20; PHQ-9; GAD-7)						
							State Flow Scale correlation state flow with self-concept HISDS correlation state flow with various well-being measures (Flourishing Scale; SWLS; ERQ; PANAS-20; PHQ-9; GAD-7) Core Flow Scale correlation core flow with self-concept HISDS correlation state flow with various well-being measures (Flourishing Scale; SWLS; ERQ; PANAS-20; PHQ-9; GAD-7)		Mean (SD) 4.02 (0.40) r = -0.10 r = between -0.40 and 0.43 Mean (SD) 4.14 (0.46) r = 0.02 r = between -0.24 and 0.32				p-value p > 0.05 p > 0.05 p > 0.05 p > 0.05
Robinson et al. (2015) [44]	Multiple sclerosis (not in-patients)	RCT (N = 56)	Balance training with exergame Nintendo Wii FIT™ Randomization in: Group1: balance training using the Nintendo Wii FIT™ (exergaming) (n = 20) Group 2: traditional balance training groups (non-exergaming) (n = 18) Group 3: control group no intervention (n = 18) Duration: 4 weeks of twice weekly 40–60 min exercise sessions	52.6 (6.1) 14 females 6 males 53.9 (6.5) 12 females 6 males 519 (4.7) 12 females 6 males	Postural sway (using a force plate), gait (GAITRite™), technology acceptance (UTAUT)	Flow State Scale (FSS)	Flow scores on the level of certain subscales were significantly higher in the Wii Fit™ as compared to control group:						
							Flow Subscale Autotelic experience Clear goals Challenge-skill balance Concentration on Task Paradox of Control Unambiguous Feedback Action Awareness Merging Transformation of Time Loss of self-Consciousness	Wii Fit™ Mean (SD) 4.6 (0.6) 4.3 (0.6) 3.9 (0.5) 4.4 (0.7) 3.8 (0.7) 4.2 (0.7) 3.9 (0.9) 4.1 (0.9) 4.3 (0.7)			Control Mean (SD) 4.1 (0.8) 4.0 (0.8) 4.2 (0.7) 3.9 (0.8) 3.9 (0.8) 3.9 (1.2) 3.3 (0.7) 2.2 (0.9) 4.3 (0.9)	p-value 0.08 0.05* 0.35 0.03* 0.17 0.04* 0.03* 0.001* 0.23	
Yoshida et al. (2018) [48]	Traumatic Brain Injury at least 6 months post-injury (not in-patients)	RCT (N = 20)	Attentional training with Video game task Randomization in: flow group (n = 10) or control group (n = 10) Patients performed a video game task, one inducing flow (flow group) and the other not (control group) for 4 weeks	41.7 (9.37) 4 females 16 males	SDMT, TMT, PASAT, Continuous Performance Test X task (CPT-X) Moss Attention Rating Scale (MARS)	Flow State Scale for Occupational Tasks (FSSOT)	Flow scores were significantly higher in the intervention group than in the control group. Both groups showed a positive, but non-significant correlation between and the FSSOT and composite score of the attention tests (TMT, SDMT, PASAT) (Flow: r = .456, p = 0.21; Control r = 0.554, p = 0.09) No significant correlation between the FSSOT and the overall MARS score (r = -0.28, p = 0.24). A significant correlation was found between one subscale of MARS (sustained/consistent) and FSSOT (r = -0.51, p < 0.05)						
Ku et al. (2018) [46]	Subacute to chronic Stroke	RCT (N = 20)	Hand wrist and foot ankle exercise with Mobile Game play Randomization in: Game based-NMES (n = 9) or Conventional-NMES (n = 11) 20 min per day for 5 consecutive days Flow measurement during each training session	MG-NMES 63.3 (10.78) 3 females 6 males C-NMES 5.1 (10.0) 5 females 6 males	No other assessment	modified version of the questionnaire from [11] 6 Flow statements (attention, curiosity and intrinsic interest	As shown, the mean scores of the Flow were > 4 points on a LIKERT scale of 1 to 5, for the MG-NMES so indicating that the patients were at high Flow level during the Mobile-Game play, as this significantly higher as compared to just C-NMES						
							attention curiosity intrinsic interest	MG-NMES Mean (SD) 4.43 (0.55) 4.11 (0.51) 4.46 (0.42)			C-NMES Mean (SD) 3.69 (0.73) 3.41 (0.86) 3.86 (0.74)	p-value 0.022* 0.044* 0.031*	
Yoshida et al. (2018) [50]	Cerebral vascular disease Orthopaedic diseases Neurodegenerative diseases Spinal cord diseases Internal diseases	RCT (N = 56)	Activities of daily living Randomization in: Experimental group (n = 28) OT with evaluating challenge-skill levels by the client and adaption within therapy Control group (n = 28) No evaluation of challenge-skill levels by the client	80.9 (8.36) 14 males 81.2 (6.51) 13 males	Health-related quality of life (EuroQol-5 Dimensions) Short-Form Health Survey for general health (SF-8)	Flow State Scale for Occupational Tasks (FSSOT)	Flow levels were on moderate level (Score ranges 14–98) for both groups. For the experimental group there was a significant difference in flow						
							FSSOT	Intervention group Mean (SD) 63.74 (11.56)			Control group Mean (SD) 54.46 (18.82)	p-value 0.008	
Yoshida et al. (2019) [49]	Cerebral, spinal and musculoskeletal diseases	RCT (N = 72)	Activities of daily living Randomization in: Experimental group (n = 36) OT with evaluating challenge-skill levels by the client and adaption within therapy Control group (n = 36) No evaluation of challenge-skill levels by the client	Experimental group 74.11 (9.11) 24 females 12 males Control group 75.17 (9.99) 21 females 15 males	Subjective Quality of life (Ikigai-9) Health-related quality of life (EuroQol-5 Dimensions, Five Levels (EQ-5D-5L)	Flow State Scale for Occupational Tasks (FSSOT)	Flow scores were at high level (Score ranges 14–98) in both groups. Higher scores for the intervention group but not significantly different between the groups						
							Intervention group Pre Post Change score Control group Pre Post Change score Effect size between the groups	FSSOT Mean (SD) 79.56 (10.9) 81.09 (10.54) 1.66 (10.15) FSSOT Mean (SD) 75.47 (15.32) 78.11 (14.15) 2.64 (10.33) -0.09					

*CFS* Core Flow Scale, *CPT-X* continuous performance test X task, *C-NMES* conventional neuromuscular electrical stimulation, *ERQ* emotion regulation questionnaire, *EQ-5D-5L* EuroQol-5 dimensions, five levels, *FSS* Flow State Scale, *FSSOT* Flow State Scale for occupational tasks, *GAD-7* generalized anxiety disorder scale, *HISDS* head injury semantic differential scale, *N* number, *nr* not reported, *MG-NMES* neuromuscular electrical stimulation, *FMA* Fugl-Meyer assessment, *MARS* moss attention rating scale, *MBI* modified Barthel Index, *Ikigai-9* subjective quality of life measurement, *PANAS-20* positive affect and negative affect scale, *PASAT* paced auditory serial addition test, *PHQ-9* patient health questionnaire-9, *RAVLT* Ray’s auditory verbal learning test, *RCT* randomized controlled trial, *SF-8* short-form health survey for general health, *SFS* Short Flow Scale, *SD* standard deviation, *SDMT* symbol digit modalities test, *TMT* trail making test, *SWLS* satisfaction with life scale, *UTAUT* unified theory of acceptance and use of technology

**Table 3:** Characteristics of the included flow questionnaires

Flow Questionnaire (Reference article)	Construct	Mode of administration	Number of items	Response options (Range)
FSS [4]	Flow State	Recall (after training)	36 items	5-point Likert 1 (strongly disagree) to 5 (strongly agree)
SFS [40]	Flow State	Recall (after training	9 items	7-point Likert 1 (strongly disagree) to 7 (strongly agree)
CFS [40]	Flow Core	Recall (after training)	10 items	5-point Likert 1 (never/strongly disagree) to 5 (always/strongly agree)
Flow in human–computer interactions [11]	Flow State	Recall (after training)	12 items	7-point Likert 1 (strongly disagree) to 7 (strongly agree)
FSSOT [41]	Flow State	Recall (after training Gameplay)	14 items	7-point Likert 1 (strongly disagree) to 7 (strongly agree)
Overall appreciation questionnaires [42]	Flow State	Recall (after training)	9 items	5-point Likert 1 (never/strongly disagree) to 5 (always/strongly agree)

*CFS* Core Flow State, *FSS* Flow State Scale, *FSSOT* Flow State Scale for occupational tasks, *SFS* Short Flow Scale

**Table 4:** Summarized results of the measurement properties of the flow questionnaires in healthy subjects

	Content Validity			Construct Validity			Reliability		Responsiveness
	Relevance	Comprehensiveness	Comprehensibility	Structural validity	Hypotheses testing	Cross-cultural validity	Internal consistency	Measurement error	
Flow in human computer interactions [11]									
(a)	−	−	−	+			+		
(b)	m			m			m		
FSS [4, 14, 53–57]									
(a)	+	+	+	+	+	+	+		
(b)	h			h	h	h	h		
FSS Greek [55]									
(a)	+	+	−	−		?	−		
(b)	l			m		l	m		
FSS Greek [56]									
(a)	+	+	+	−	−	?	+		
(b)	h			h	h	m	h		
FSS Spanish [57]									
(a)	+	+	−	−		?	?		
(b)	m			h		m	m		
SFS [40]									
(a)	+	+	−	−	+		+		
(b)	m			h	h		h		
CFS [40]									
(a)	+	+	−	−	−		−		
(b)	m			h	h		h		
FSSOT [41]									
(a)	+	+	−	+	+		+		
(b)	m			h	h		h		

*h* high, *m* moderate, *l* low, *vl* very low, *CFS* Core Flow Scale, *FSS* Flow State Scale, *FSSOT* Flow State Scale for occupational tasks, *SFS* Short Flow Scale, (a) Overall rating; (b) Quality of evidence

### Different flow questionnaires and their use in neurological diseases

The Flow State Scale (FSS) was used in patients with PD [43] and in patients with MS [44]. Baker et al. (2015) applied the Short Flow Scale (SFS) and the Core Flow Scale (CFS) [40] in patients with TBI. Van der Kuil et al. (2018) used a self-developed overall appreciation questionnaire in patients with stroke, TBI and spinal cord injury. Six items in this questionnaire were adapted from the FSS and three items were further added. The Flow State Scale for Occupational Tasks questionnaires (FSSOT) was used by Yoshida Kazuki, et al. (2014; 2018) in patients with TBI and was also used by Yoshida Ippei, et al. (2018) in patients with stroke and spinal cord injury. In contrast to these previous studies, which used known questionnaires, Shin and colleagues (2014) used six different flow questions [45] in patients with stroke, which were slightly adapted from another study done in TBI [46].

The different flow questionnaires were mainly used to get an overall impression of the flow psychological state of neurologically impaired patients when they were engaged in different training modes, such as upper limb or lower limb training in patients with stroke [45]^45^, balance training in patients with MS [44] and PD [43], cognitive training in patients with TBI [47, 48], and stroke [42]. In seven out of the ten studies, as presented in Table 2, serious games were used as therapeutic intervention. The designs of the studies were either pilot and explorative in nature, testing the usability of a new serious game [42, 43, 45, 47] or pilot Randomized Controlled Trials (RCT) evaluating the preliminary efficacy of new games [44, 46, 48].

Four usability studies measured flow in order to quantify the level of immersion into the gameplay [42, 43, 45, 47]. Shin et al. (2014) developed a task-specific interactive, game-based virtual reality rehabilitation system (RehabMaster) for the rehabilitation of the upper extremities after a stroke. During the development phase 20 stroke patients completed a six-item questionnaire adopted by [11] to test if they were engaged and if the training was a positive experience, so that they were motivated to continue. For all statements, the participants gave lower scores for the negative questions (e.g., “Using RehabMaster was boring for me”) and higher scores for the positive questions (e.g., “RehabMaster was fun for me to use”) on a 5-point Likert Scale [45]. The participants indicated that the RehabMaster-based training and games maintained their attention, were enjoyable and without eliciting any negative feelings [45]. Galna et al. (2014) developed a computer game to rehabilitate dynamic postural control for patients with PD using the Microsoft Kinect. Also, during the pilot phase, flow experience was recorded from nine participants with PD by means of the FSS questionnaire. The FSS was rated on a 5-point Likert Scale. The flow subscales “concentration” showed the highest mean value across the participants (Mean 4.56), followed by high scores of the subscales “loss of self-consciousness” (Mean 4.14), clear goals (Mean 4.22) and enjoyment (Mean 4.03). Lower flow scores were found in the subscale “transience” (Mean 2.67) and action-awareness (Mean 3.11). Van der Kuil et al. (2018) designed a cognitive rehabilitation therapy for patients with acquired brain injuries in form of a serious game. The aim of the serious game was to aid patients in the development of compensatory navigation strategies by providing exercises in 3D virtual environments on their home computers. During the testing of the software application, questions about the general appreciation were asked at the beginning and at the end of the experimental phase. Van der Kuil et al. (2018) constructed an “overall appreciation questionnaire” of nine items rated on a 5-point Likert scale. Six items were adapted from the FSS and three items were constructed in the context of a usability test. The highest scores were found in the “attention” (Mean 4.79) and “concentration” items (Mean 4.54). The item “control” presented the lowest score (Mean 3.29). Yoshida K. et al. (2014) conducted an exploratory case study with two patients with attention-deficit disorder after TBI. Two types of video game tasks for attention training were created. The first type of video game was balancing levels of skill and challenge and gave quick feedback about the score. In the second type of video game, the level of the difficulty of the task was constant and the participant received no information about the goal or a score feedback. Patient A performed the first type of video game for 14 days after receiving general occupational therapy for 11 days. Patient B performed the first type of video game for 15 days after performing the second type of video game for 10 days. The FSSOT was administered to identify the patient’s flow state. The results for Patient A suggested that the first type of video game was more effective than general occupational therapy for improving attention deficits. The results for Patient B suggested that the first type of video game was more effective than the second type of video game.

Five RCTs measured flow in intervention groups and in control groups. Three RCTs used video games and actually compared levels of flow between the intervention and control group (Wii Fit™ vs. traditional balance training in patients with MS [44]; or Mobile Game—Neuromuscular Electrical Stimulation (NMES) vs. Conventional NMES in patients with stroke [46] and Yoshida K. et al. (2018) compared flow in an attention gameplay intervention in patients with traumatic brain injuries. In Robinson et al. (2015) the intervention group that trained balance with Wii Fit™ showed significantly higher flow scores in the flow subscales clear goals (p = 0.05), concentration on the task (p = 0.03), unambiguous feedback (p = 0.04), action awareness merging (p = 0.03) and transformation of time (p = 0.001) than the control group [44]. Likewise, the hand-wrist and foot–ankle training with serious games presented significantly higher scores in attention (p < 0.05), curiosity (p < 0.05) and intrinsic interest (p < 0.05) compared to the control group which was not playing serious games [46]. Both previous RCT’s focused on videogames based on physical training, whereas the third RCT by Yoshida K. et al. (2018) investigated flow during cognitive training. They examined whether the intervention group during a serious game for attentional training by adapting the challenge to the patient’s skill, gave clear goals and prompt feedback about the score. The level of the difficulty of the task was constant in the control group and they received no information about the goal or score feedback. The study population in this RCT had a traumatic brain injury at least 6 months ago. The researchers stated that the FSSOT score was significantly higher in the intervention group than in the control group. Both groups showed a positive association between the increase in the composite score of the attention tests [Trail Making Test (TMT), Symbol Digit Modalities Test (SDMT), Paced Auditory Serial Addition Test (PASAT)] and the FSSOT score. Although the correlation coefficients presented a large effect, the correlations were not significant (Flow: r = 0.456, p = 0.21; Control r = 0.554, p = 0.9). The total of the Moss attention rating scale (MARS) demonstrated no association with the FSSOT score, except one subitem that obtained a significant negative correlation (sustained/consistent attention, r = 0.51, p < 0.05). Two RCT’s by Yoshida I. et al. (2018; 2019) did not use videogame-based training but consciously adapted the challenge to the abilities during occupational therapy (OT) in patients with cerebral, spinal disease [49] and older adults with various neurological disease [50]. Attention was paid to an optimal challenge-skill balance when performing activities of daily living (ADLs) such as eating, laundry, cooking, shopping, etc. The training was adapted so that in the interventions group the participants and the therapists quantified and shared the task performance based on a scale of challenges and skills and adjusted the requirements for the task accordingly. On the other hand, in the control group the challenge-skill of the trained ADLs was not adjusted over the training sessions. In the 2018 paper there were 10 sessions, once a week and training focused on just one activity, evaluated and selected after filling out the Canadian Occupational Performance Measure (COPM) [51]. The COPM is a personalized, client-centred instrument designed to identify the occupational performance problems experienced by the client. Using a semi-structured interview, the therapist initiates the COPM process by engaging the client in identifying daily occupations of importance that they either want to do, need to do, or are expected to do but are unable to accomplish [51]. In the 2019 study, the participants selected not one, but several ADLs based on the outcome of the COPM as treatment goals. Treatments in each group comprised sessions lasting 40–60 min, conducted six times per week. In both RCT’s flow experience was measured pre- and post-treatments with the FSSOT. In the first RCT [50] there was a highly significant interaction effect for flow (p = 0.008, d = 0.82), in favour of the adjusted challenge-skill OT, as compared with the control group. This interaction was not confirmed in their follow-up study (p > 0.05, d = 0.31) [49].

Similar to Yoshida I. (2018, 2019), Baker et al. (2015) also did not use videogame based training but explored if song writing interventions for patients with TBI and spinal cord injuries in the early phase of neurorehabilitation would support a change in self-concept and well-being [52]. By means of a non-randomized repeated measures design, they found that flow scores were very high after the intervention. However, these scores did not significantly correlate with self-concept Head Injury Semantic Differential Scale (HISDS) (State Flow Scale r = − 0.10; p > 0.05; Core Flow Scale r = 0.02; p > 0.05) nor with 7 different well-being measures evaluating sense of flourishing, life satisfaction, coping, affect, depression, and anxiety (State Flow Scale r = between − 0.40 and 0.43; p > 0.05; Core Flow Scale r = between − 0.24 and 0.32; p > 0.05).

### Psychometric properties of flow questionnaires

The Summary of Findings (SoF) per measurement property, its overall rating and the grading of the quality of evidence are presented in Table 4. The COMSIN guidelines [27] were applied for the rating of the SoF and were as following: [Overall Rating: sufficient (+), insufficient (−), undetermined (?); Quality of Evidence high (h), moderate (m), low (l), very low (lw)]. If a measurement property was not analysed or not reported, the rating box remains empty. The rating criteria for good measurement properties and for the quality of evidence are presented in the Additional file 2.

### Content validity

Content validity including relevance, comprehensiveness and comprehensibility was assessed for the FSS and for FSSOT. Jackson et al. conducted two qualitative studies with elite athletes [58, 59] prior to the development of the FSS. The SFS and CFS were also developed by the Jackson Group with the intention of creating a short version of the FSS and DFS, respectively. Yoshida K. et al. (2013) tested the FSSOT in the development phase by experts on flow theory. Both Jackson et al. (1996) and Yoshida K. et al. (2013) conducted pilot-testing before the validation procedure.

### Structural validity

Structural validity, by means of confirmatory and internal consistency was determined in all flow questionnaires. All studies presented good internal consistency (Cronbach alpha above 0.70). Confirmatory factory analysis was performed in all flow questionnaires. Taking the strict COSMIN guidelines [27] into account the CFS questionnaire fulfilled the parameters requested by the COSMIN guidelines (CFI or TLI > 0.95 OR RMSEA < 0.06 OR SRMR < 0.08), the SFS, FSS and FSSOT had parameters approaching closely these cut-offs, so validating high quality of evidence. The questionnaire by Webster et al. (1993) showed considerably lower scores, pinpointing to moderate quality of evidence.

### Cross-cultural validity

The FSS was cross-culturally validated in Greek [55, 56] and in Spanish [57]. They all followed standard back and forward translation procedures. Stavrou and Zervas (2004) tested a second FSS-Greek version, since the first one done by Doganis et al. (2002) indicated rather a moderately fit to the data, whereas the internal consistency (Cronbach alpha) was below 0.70 for some of the FSS subscales (action-awareness merging = 0.34, concentration on task at hand = 0.64, transformation of time = 0.67). The FSS-Greek version by Stavrou and Zervas (2004) presented an internal structure validity ranging from Cronbach alpha of 0.75 to 0.92 (mean = 0.82) and a closely fit to the cut-off’s parameters requested by the COSMIN guidelines. The Spanish version of the FSS presented a good internal consistency (Cronbach alpha above 0.70) and the structural validity was tested with a confirmatory factory analysis, demonstrating a close fit to the cut-offs parameters [57].

### Construct validity

Construct validity, by means of convergent validity, was assessed for the FSSOT total scores, showing significant negative correlations with the total score of State-Trait Anxiety Inventory (STAI) (r = − 0.537, p < 0.01) [41]. Jackson et al. (1998) examined psychological correlates of state flow in a separate study than the original validation paper [4]. Significant associations were found between the variables FSS total and perceived athletic ability (PSA) (r = 0.33, p < 0.01); total anxiety (A-SUM) (r = − 0.34, p < 0.01) and intrinsic motivation to experience stimulation (IMSTIM) (r = 0.25, p < 0.01). A series of external validity analyses was conducted for the SFS and CFS by Martin et al. (2008) for each subdomain “work”, “sport” and “music” in SFS and “general school”, “mathematics” and “extracurricular” in CFS with the Motivation and Engagement Scale (MES), which includes the following key correlates: participation (SFS: mean r 0.74–0.90; CFS: mean r 0.25–0.56), enjoyment (SFS: mean r 0.73–0.89); CFS mean r 0.13–0.71), buoyancy (SFS: mean r 0.68–0.81; CFS: mean r 0.15–0.42), aspirations (SFS: mean r 0.71–0.81; CFS: mean r 0.12–0.68), adaptive cognitions (SFS: mean r 0.72–0.82; CFS: mean r 0.23–0.74), adaptive behaviours (SFS: mean r 0.59–0.70; CFS: mean r 0.18–0.83), impeding/maladaptive cognitions (SFS: mean r − 0.37 to − 0.59; CFS: mean r − 0.10 to − 0.23), and maladaptive behaviours (SFS: mean r − 0.47 to − 70; CFS: mean r − 0.15 to − 0.79). The SFS presents higher correlations with the MES than the CFS. Significance of the correlations was not reported.

### Reliability

None of the identified studies investigated reliability (test–retest), measurement error, criterion validity or responsiveness of the flow questionnaires. As far as we know, none of the flow questionnaires have been tested for their psychometric properties in neurologically impaired people.

### Interpretability and feasibility of the included flow questionnaires

Floor and ceiling effects, completion time and costs of instrument and contact information of used outcomes measuring flow are listed in Table 5.

**Table 5:** Interpretability and feasibility of the included flow questionnaires

PROM	Floor and ceiling effects	Completion time	Copyright	Costs of instrument	Contact information
Flow in human–computer interactions	nr	A couple of min	No copyright	Free to use	Appendix A of [11]
FSS	nr	10 Min	© 2010 by Susan A. Jackson	Manual $50.00 $ 2.50 per questionnaire (minimum purchase of 20 questionnaires)	https://www.mindgarden.com/100-Flow-scales
SFS	nr	5 Min	© 2010 by Susan A. Jackson	Manual $50.00 $ 2.50 per questionnaire (minimum purchase of 20 questionnaires)	https://www.mindgarden.com/100-Flow-scales
CFS	nr	5 Min	© 2010 by Susan A. Jackson	Manual $50.00 $ 2.50 per questionnaire (minimum purchase of 20 questionnaires)	https://www.mindgarden.com/100-Flow-scales
FSSOT	2 items with ceiling effect were removed, no floor effect	2 Min	© 2013 by Kazuki Yoshida	Free in scientific research	Appendix of [41]

*CFS* Core Flow State, *FSS* Flow State Scale, *FSSOT* Flow State Scale for occupational tasks, *nr* not reported, *SFS* Short Flow Scale

## Discussion

The aim of the present study was to identify and systematically review current literature on flow experience assessed in patients with neurological diseases such as stroke, TBI, MS and PD. In addition, we critically appraised, compared and summarized the measurement properties of self-reported flow questionnaires used in a neurorehabilitation setting.

Flow experience in patients with neurological disorders has so far been measured in only a few studies, some of them very pilot in nature, being usability studies, other were RCTs, and mostly related to serious gaming [42–45, 47, 48]. One aim of such interventions is to achieve an optimal flow state of the patient, possibly creating an optimal learning environment to improve either physical and/or cognitive functions (being for example improving balance, or attention). Flow questionnaires are one way to capture this flow state, since the patient is, immediately after the intervention, asked for his or her experiences. In this way, the clinician gets an overall impression whether the patient was in an optimal psychological state of flow or not. Our systematic review demonstrated that six flow questionnaires were used so far.

However, psychometric properties of these questionnaires were established only in athletes and other healthy populations so far, and not in neurologically impaired patients. Latter population often suffer from cognitive problems (disturbed vigilance, working memory deficits, language comprehension difficulties) which may impact the assessment of flow.

The FSS and FSSOT appear to be good candidate questionnaires, based on their good psychometric validity properties in healthy subjects. The FSSOT, compared to the FSS, requires less administration time so probably being more feasible for neurologically impaired patients, taking mild cognitive deficits into account. Besides proper validation, reliability measures such as test–retest, measurement errors will have to be established as well because these reliability measures give an overall impression about the stability of item responses. A final aspect will be to evaluate the internal (the ability to measure change over time) and external responsiveness (the extent to which changes in a measure relate to corresponding changes in a reference measure) of these flow questionnaires. Only when these psychometric properties are well defined the outcome of flow questionnaires can be better interpreted in either usability studies or RCT’s.

The investigation of flow experience in neurological patients started at about the same time as the development of serious games for rehabilitation therapy. The integration of motivational strategies in the form of “gamification” is one of the benefits of the new therapy options [19, 60]. The expectation of such therapy programs is that they will strengthen compliance with repetitive high-dose functional training programs [19, 60]. The game developer's aim is to bring the patient into a flow state that leads to an optimal gaming experience [61]. They expect to foster the engagement through the gamification of the therapeutic exercises and at the same time give the therapist the possibility to control and customize the levels of complexity of the rehabilitation training. Seven of the ten included studies measured flow experience in the context of serious games in patients with stroke, PD, MS and/or TBI [42–48]. Flow experience was mainly assessed in the context of usability studies in newly developed serious game therapy programs for rehabilitation purposes [42, 43, 45, 47]. Our review showed that total flow mean scores between 3.76 and 4.33 points on a 5-point Likert scale were achieved in all studies when serious games were used as physical-therapeutic exercises [42–46] compared to control groups without serious games, these flow mean points reached 3.65–3.76 [44, 46]. It turns out that therapeutic interventions with a game-like character stimulate concentration and enjoyment. This assumption was substantiated as flow experience was higher in game therapy versus conventional therapy, shown in two intervention studies investigating balance with Wii FIT™ [44] and hand wrist, foot ankle training with serious games [46] (Table 1). An advantage of rehabilitation therapy with a game character is that the goals and the rules of the task of the game are clearly defined. In addition, players receive immediate feedback of performance as to whether the task was performed correctly or not, a key element of the motor learning theory [57]. This, in turn, allows the movements to be deliberately adjusted in line with performance. If these components are appropriate, this also has a positive effect on concentration. In the principles of motor learning, feedback, but also the ability to concentrate on a task, and the motivation to perform an exercise, are essential for learning new motor skills [62, 63]. Therefore, we assume that positive flow experiences during physical exercises support motor learning. From this perspective, it makes sense to measure flow experience in the development and testing phase of new therapy games. In this way it is possible to determine which adjustments should be made, e.g., to define the goal or the rules of the application more precisely.

Whether flow experiences ultimately had a positive effect on the physical outcome parameters was not investigated in these studies. Three studies from Japan explored in TBI patients and older adults with various neurological diseases whether flow experience had an effect on attention [48] and health related quality of life [49, 50]. In a small RCT (n = 20), Yoshida K. et al. (2018) created two types of attention demanding serious games exercises, the flow task and the control task. The control task maintained a constant level of task difficulty regardless of the patient’s skill and did not give any goal and feedback about the score. Both tasks had identical content, except that the flow task was designed to induce flow by increasing task difficulty according to patients’ skill and giving clear goals and quick feedback about the score. Yoshida and colleagues (2018) referred to the Flow Theory of Nakamura and Csikszentmihalyi (2009), suggesting that three key characteristics of the flow theory (challenge-skill balance, clear goals and feedback) are essential to generate flow experience and that these characteristics are externally controllable. They found significantly (p-value not reported in the paper) higher flow total values in the intervention group (flow task) compared to the control group (control task) [48], suggesting that the way a serious game is designed, with regard to its task difficulty, can positively affect the flow state of a patient. Both groups showed a positive, but non-significant association between the increase in the composite score of cognitive attention tests (TMT, SDMT, PASAT) and the FSSOT total score (Flow: r = 0.456, p = 0.21; Control r = 0.554, p = 0.9) [48]. The lack of significant correlation, between attention and flow test scores may be explained by the pilot nature and small sample size of this RCT. Regardless, the fact that the flow psychological state was amenable to task difficulty gave a first indication that the state of flow may facilitate training, being worthwhile to investigate in further studies.

In two larger RCT’s, both conducted by Yoshida I. (2018, 2019), the outcomes of both RCT’s differed regarding the effect of the training on flow. While in their first RCT significant effects on flow, in favour of the experimental OT were found, this was not the case in their follow-up RCT. The reason for this discrepancy may be twofold. Firstly, in their first RCT the focus was on one activity and not on multiple ADLs, as in their second RCT. Presumably, in a rehabilitation setting, the focus is on improving the skills of one activity at a time rather than several at once. Therefore, it may be easier for participants to experience flow. For achievement of performance competence is a process that takes time, practice, and thorough skill development until the optimal performance of the skill (referred to as mastery) is characterized by an obvious ease and grace [2]. According to Flow-theory, to attain this state, an optimal balance between challenge and skill during training is crucial [36, 49]. This is because anxiety is experienced when challenge exceeds ability, and boredom is experienced when ability exceeds challenge. Thus, it can be said that the better the challenge is matched to the ability and the expertise in performing is increasing, the easier it is to experience flow, as shown in other studies [6, 7, 64]. The second reason may lie in the much higher baseline flow levels the patients had in the second RCT, as compared with the flow levels of the patients in the first RCT, therefore leaving almost no room for further improvement. Irrespective of the discrepancy of results between both RCTs, the fact that patients could improve their flow by means of an adjusted challenge-skill OT training, by focusing on one specific ADL task is promising. One could explore, in future studies, for example the effects of improved flow on upper limb skills by doing challenge-skill ADL training, and this in different contexts, so the patient gets into high levels of flow.

Six different flow questionnaires were applied in these studies, leaving the question open which one to be taken for future validation in neurologically impaired patients. Based on their good psychometric properties in healthy subjects, both FSS and the FSSOT seem to be good candidates. The flow questions in the FSS are strongly related to concepts in the field of sport, and its administration time is rather long, (36 items). Therefore, feasibility might be questionable, especially if one considers the rather busy schedules of clinicians working in neurorehabilitation facilities. Subsequent shorter versions of the FSS were developed, being the SFS and CFS [40]. Still, the authors do recommend combining these measures when evaluating flow, which may be impractical. Furthermore, the flow questions are still very much related to the context of sport psychology, and less for neurorehabilitation purposes. This might also explain why, for example, Van der Kuil et al. (2018), for their study in patients with acquired brain injury, used 6 items of the FSS and then adapted them content wise, to make it more comprehensible and applicable for these patients’ group.

With regard to the FSSOT, its 14-item length seems more feasible as compared to the longer FSS. Furthermore, having been used already in two RCT’s to assess flow experience after challenge-skills based ADL training [49, 50] and in one RCT to assess flow experience in attentional training in patients with neurological impairments [48], this questionnaire seems to be best candidate, and worthwhile to be properly validated in these patient groups. Depending on other contexts, such as upper limb virtual reality or robotic-assisted training, the questions of the FSSOT can be further adapted in the light of different cultural backgrounds.

### Limitation

A possible limitation of this review was that we could not present a quality assessment of study design, since both exploratory, non-randomized as well as randomized trials were included. Another limitation is that we included studies in patients with various neurological disorders that affect overall study population homogeneity. Hence, one has to be careful in comparing the results of these studies directly. Finally, publication bias may be present, as well as a language bias, given that we considered only flow questionnaires described in predefined databases and restricted our search to English language publications.

## Conclusion

To sum up, the present review indicates that flow experience is increasingly measured in the physical/cognitive rehabilitation setting in patients with neurological disease such as stroke, TBI, MS and PD. Flow experience was mainly measured immediately after a therapeutic intervention that aimed to improve physical or cognitive functions with serious exergaming. In seven out of ten studies in which new games for therapy were developed, patients flow experience was measured to find out to what extent they were engaged to the new games [42–48]. The other three studies assessed flow during occupational therapy when practicing ADL’s [49, 50] and during music therapy [52]. Six different flow questionnaires were applied in these studies. None were specifically validated in patients with neurological diseases. Therefore, the psychometric properties of used tests for measuring flow experience are lacking and will have to be evaluated in future studies. For exergame developers working in the field of physical/cognitive rehabilitation in patients with neurological diseases, a valid flow questionnaire can help to further optimize the content of the games so that optimal engagement can occur during the gameplay.

## Supplementary Information

**Additional file 1.** Search strategy.

**Additional file 2.** Methodology quality and results of flow questionnaires per measurement properties and the rating criteria for good measurement properties.

## Data Availability

All data generated or analysed during this study are included in the published article.

## Abbreviations

ADL: Activity of daily living
A-SUM: Total anxiety
CFI: Comparative fit index
CFS: Core Flow Scale
COPM: Canadian occupational performance measure
COSMIN: Consensus-based standards for the selection of health measurement instrument
FMA: Fugl-Meyer assessment
FSS: Flow State Scale
FSSOT: Flow State Scale occupational task
GRADE: Grading of recommendations assessment, development, and evaluation
HISDS: Head injury semantic differential scale
IMSTIM: Intrinsic motivation to experience stimulation
M: Mean
MBI: Modified Barthel Index
MES: Motivation and engagement scale
MeSH: Medical subject headings
MS: Multiple sclerosis
N: Number
NMES: Neuromuscular electrical stimulation
nr: Not reported
OT: Occupational therapy
PASAT: Paced auditory serial addition test
PD: Parkinson’s disease
PICOS: Participants, intervention, comparison, outcome, study design framework
PRISMA: Preferred reporting items for systematic reviews and meta-analyses statement
PROM: Patients (or participants)-reported outcome measures
PROSPERO: International prospective register of systematic review
PSA: Perceived athletic ability
RAVLT: Ray’s auditory verbal learning test
RCT: Randomized controlled trial
RMSEA: Root mean square error of approximation
SD: Standard deviation
SDMT: Symbol digit modalities test
SF-8: Short-form health survey for general health
SFS: Short Flow Scale
SoF: Summary of findings
SRMR: Standardized root mean square residual
STAI: State-Trait anxiety inventory
TBI: Traumatic brain injury
TLI: Tucker-Lewis Index
TMT: Trail-making test
UE: Upper extremity
UTAUT: Unified theory of acceptance and use of technology
VR: Virtual reality
X^2^: Chi-Square

## References

[1] Csikszentmihalyi M (1975). Beyond boredom and anxiety.

[2] Csikszentmihalyi M. Flow and the foundations of positive psychology. Flow and the foundations of positive psychology. 2014.

[3] Nakamura J, Csikszentmihalyi M. The concept of flow. In: Oxford handbook of positive psychology. 2009. p. 195–206.

[4] Jackson SA, Marsh HW (1996). Development and validation of a scale to measure optimal experience: the flow state scale. J Sport Exerc Psychol.

[5] Abuhamdeh S (2020). Investigating the “Flow” experience: key conceptual and operational issues. Front Psychol.

[6] Jackson SA, Thomas PR, Marsh HW, Smethurst CJ (2001). Relationships between flow, self-concept, psychological skills, and performance. J Appl Sport Psychol.

[7] Engeser S, Rheinberg F (2008). Flow, performance and moderators of challenge-skill balance. Motiv Emot.

[8] Koehn SMT (2012). The relationship between performance and flow state in tennis competition. J Sport Med Phys Fit.

[9] Perttula A, Kiili K, Lindstedt A, Tuomi P (2017). Flow experience in game based learning—a systematic literature review. Int J Serious Games.

[10] Stavrou NA, Jackson SA, Zervas Y, Karteroliotis K (2007). Flow experience and athletes’ performance with reference to the orthogonal model of flow. Sport Psychol.

[11] Webster J, Klebe Trevino L, Ryan L. J human behavior. Comput Human Behav. 1993;9(1):411–26.

[12] Delle Fave A, Massimini F, Bassi M. Instruments and methods in flow research. In: Psychological selection and optimal experience across cultures advancements in positive psychology 2. 2011. p. 59–87.

[13] Swann CF. Flow in sport. In: Flow experience: empirical research and applications. 2016. p. 51–64.

[14] Marsh HW, Jackson SA (1999). Flow experience in sport: construct validation of multidimensional, hierarchical state and trait responses. Struct Equ Model.

[15] Kiili K (2006). Evaluations of an experiential gaming model. Hum Technol Interdiscip J Humans ICT Environ.

[16] Procci K, Singer AR, Levy KR, Bowers C (2012). Measuring the flow experience of gamers: an evaluation of the DFS-2. Comput Human Behav [Internet]..

[17] Zhang J, Fang X, Chan SS (2013). Development of an instrument for studying flow in computer game play. Int J Hum Comput Interact.

[18] Laver K, Lange B, George S, Deutsch J, Saposnik G, Crotty M. Virtual reality for stroke rehabilitation. Cochran Database Syst Rev. 2017;(11). 10.1002/14651858.CD008349.pub4 PMC6485957 29156493

[19] O’Neil O, Fernandez MM, Herzog J, Beorchia M, Gower V, Gramatica F (2018). Virtual reality for neurorehabilitation: insights from 3 European clinics. PM R.

[20] van Beek JJ, van Wegen EE, Bohlhalter S, Vanbellingen T (2019). Exergaming-based dexterity training in persons with Parkinson disease: a pilot feasibility Study. J Neurol Phys Ther.

[21] Nef T, Chesham A, Schütz N, Botros AA, Vanbellingen T, Burgunder J, et al. Development and evaluation of maze-like puzzle games to assess cognitive and motor function in aging and neurodegenerative diseases. 2020;12(April). 10.3389/fnagi.2020.00087 PMC7188385 32372942

[22] Ma M, Zheng H, Brahnam S, Jain L (2011). Virtual reality and serious games in healthcare. Adv comput intell paradigms in healthcare.

[23] Swanson LR, Whittinghill DM (2015). Intrinsic or extrinsic? Using videogames to motivate stroke survivors: a systematic review. Games Health J.

[24] Barbosa H, Castro AV, Carrapatoso E (2018). Serious games and rehabilitation for elderly adults. GSJ.

[25] Baur K, Schättin A, De Bruin ED, Riener R, Duarte JE, Wolf P. Trends in robot-assisted and virtual reality-assisted neuromuscular therapy: a systematic review of health-related multiplayer games. J Neuroeng Rehabil. 2018;15(1). 10.1186/s12984-018-0449-9 PMC6245892 30454009

[26] Liberati A, Altman DG, Tetzlaff J, Mulrow C, Gøtzsche PC, Ioannidis JPA, et al. The PRISMA statement for reporting systematic reviews and meta-analyses of studies that evaluate health care interventions: explanation and elaboration. PLoS Med. 2009;6(7). 10.1371/journal.pmed.1000100 PMC2707010 19621070

[27] Prinsen CAC, Mokkink LB, Bouter LM, Alonso J, Patrick DL, de Vet HCW (2018). COSMIN guideline for systematic reviews of patient-reported outcome measures. Qual Life Res [Internet]..

[28] Ottiger B, Van Wegen E, Sigrist K, Nef T, Nyffeler T, Kwakkel G, et al. Getting into a “Flow” state: a systematic review of flow experience in neurological disease. PROSPERO [Internet]. 2020;CRD4202018. https://www.crd.york.ac.uk/prospero/display_record.php?ID=CRD42020187510. 10.1186/s12984-021-00864-w PMC8059246 33879182

[29] Foletto AA, d’Ornellas MC, Prado ALC (2017). Serious games for Parkinson’s disease fine motor skills rehabilitation using natural interfaces. Stud Health Technol Inform.

[30] Shirzad N, Van Der Loos HFM (2016). Evaluating the user experience of exercising reaching motions with a robot that predicts desired movement difficulty. J Mot Behav.

[31] Belchior P, Marsiske M, Sisco S, Anna Yam WM (2012). Older adults’ engagement with a video game training program, activities, adaptation & aging. Act Adapt Aging..

[32] Barry G, van Schaik P, MacSween A, Dixon J, Martin D (2016). Exergaming (XBOX Kinect^TM^) versus traditional gym-based exercise for postural control, flow and technology acceptance in healthy adults: a randomised controlled trial. BMC Sports Sci Med Rehabil [Internet]..

[33] Pedroli E, Greci L, Colombo D, Serino S, Cipresso P, Arlati S, Mondellini M, Boilini L, Giussani V, Goulene K, Agostoni M, Sacco M, Stramba-Badiale M, Giuseppe Riva AG (2018). Characteristics, usability, and users experience of a system combining cognitive and physical therapy in a virtual environment: positive bike. Sensors..

[34] de Sampaio Barros MF, Araújo-Moreira FM, Trevelin LC, Radel R (2018). Flow experience and the mobilization of attentional resources. Cogn Affect Behav Neurosci.

[35] Kawabata M (2018). Facilitating flow experience in physical education settings. Psychol Sport Exerc.

[36] Yoshida I, Hirao K, Kobayashi R (2018). Effect of adjusting the challenge-skill balance for occupational therapy: study protocol for a randomised controlled trial. BMJ Open.

[37] Thomas S, Fazakarley L, Thomas PW, Collyer S, Brenton S, Perring S (2017). Mii-vitaliSe: a pilot randomised controlled trial of a home gaming system (Nintendo Wii) to increase activity levels, vitality and well-being in people with multiple sclerosis. BMJ Open.

[38] Esfahlani SS, Thompson T, Parsa AD, Brown I, Cirstea S (2018). ReHabgame: a non-immersive virtual reality rehabilitation system with applications in neuroscience. Heliyon [Internet].

[39] Lin CS, Jeng MY, Yeh TM. The elderly perceived meanings and values of virtual reality leisure activities: a means-end chain approach. Int J Environ Res Public Health. 2018;15(4). 10.3390/ijerph15040663 PMC5923705 29614012

[40] Martin AJ, Jackson SA (2008). Brief approaches to assessing task absorption and enhanced subjective experience: examining “short” and “core” flow in diverse performance domains. Motiv Emot.

[41] Yoshida K, Asakawa K, Yamauchi T, Sakuraba S, Sawamura D, Murakami Y (2013). The flow state scale for occupational tasks: development, reliability, and validity. Hong Kong J Occup Ther [Internet].

[42] van der Kuil MNA, Visser-Meily JMA, Evers AWM, van der Ham IJM (2018). A usability study of a serious game in cognitive rehabilitation: a compensatory navigation training in acquired brain injury patients. Front Psychol..

[43] Galna B, Jackson D, Schofield G, McNaney R, Webster M, Barry G (2014). Retraining function in people with Parkinson’s disease using the Microsoft kinect: game design and pilot testing. J Neuroeng Rehabil.

[44] Robinson J, Dixon J, Macsween A, van Schaik P, Martin D (2015). The effects of exergaming on balance, gait, technology acceptance and flow experience in people with multiple sclerosis: a randomized controlled trial. BMC Sports Sci Med Rehabil.

[45] Shin JH, Ryu H, Jang SH (2014). A task-specific interactive game-based virtual reality rehabilitation system for patients with stroke: a usability test and two clinical experiments. J Neuroeng Rehabil.

[46] Ku J, Lim T, Han Y, Kang YJ (2018). Mobile game induces active engagement on neuromuscular electrical stimulation training in patients with stroke. Cyberpsychol, Behav Soc Netw.

[47] Yoshida K, Sawamura D, Ogawa K, Ikoma K, Asakawa K, Yamauchi T (2014). Flow experience during attentional training improves cognitive functions in patients with traumatic brain injury: an exploratory case study. Hong Kong J Occup Ther.

[48] Yoshida K, Ogawa K, Mototani T, Inagaki Y, Sawamura D, Ikoma K, et al. Flow experience enhances the effectiveness of attentional training: a pilot randomized controlled trial of patients with attention deficits after traumatic brain injury. NeuroRehabilitation [Internet]. 2018;43(2):183–93. http://hdl.handle.net/2115/71654. 10.3233/NRE-172396 30040757

[49] Yoshida I, Hirao K, Kobayashi R (2019). The effect on subjective quality of life of occupational therapy based on adjusting the challenge–skill balance: a randomized controlled trial. Clin Rehabil.

[50] Yoshida I, Hirao K, Nonaka T (2018). Adjusting challenge-skill balance to improve quality of life in older adults: a randomized controlled trial. Am J Occup Ther.

[51] Law M, Baptiste S, Mccoll M, Opzoomer A, Polatajko H, Pollock N (1990). The Canadian occupational performance measure: an outcome measure for occupational therapy. Can J Occup Ther.

[52] Baker FA, Rickard N, Tamplin J, Roddy C (2015). Flow and meaningfulness as mechanisms of change in self-concept and well-being following a songwriting intervention for people in the early phase of neurorehabilitation. Front Hum Neurosci.

[53] Jackson SA, Ford SK, Kimiecik JC, Marsh HW (1998). Psychological correlates of flow in sport. J Sport Exerc Psychol.

[54] Vlachopoulos SP, Karageorghis CI, Terry PC (2000). Hierarchical confirmatory factor analysis of the Flow State Scale in exercise. J Sport Sci.

[55] Doganis G, Iosifidou P, Vlachopoulos S (2000). Factor structure and internal consistency of the Greek version of the Flow State Scale. Percept Mot Skills.

[56] Stavrou NA, Zervas Y (2004). Confirmatory factor analysis of the Flow State Scale in sports. Int J Sport Exerc Psychol.

[57] García Calvo T, Jiménez Castro R, Santos-Rosa Ruano FJ, Reina Vaíllo R, Cervelló GE (2008). Psychometric properties of the Spanish version of the Flow State Scale. Span J Psychol.

[58] Jackson SA (1992). Athletes in flow: a qualitative investigation of flow states in elite figure skaters. J Appl Sport Psychol.

[59] Jackson SA (1995). Factors influencing the occurrence of flow state in elite athletes. J Appl Sport Psychol.

[60] van der Kooij K, van Dijsseldonk R, van Veen M, Steenbrink F, de Weerd C, Overvliet KE (2019). Gamification as a sustainable source of enjoyment during balance and gait exercises. Front Psychol..

[61] Kiili K (2005). Content creation challenges and flow experience in educational games: the IT-Emperor case. Internet High Educ.

[62] Cirstea CM, Ptito A, Levin MF (2006). Feedback and cognition in arm motor skill reacquisition after stroke. Stroke.

[63] Kitago T, Krakauer JW (2013). Motor learning principles for neurorehabilitation. Handb Clin Neurol.

[64] Stavrou NAM, Psychountaki M, Georgiadis E, Karteroliotis K, Zervas Y (2015). Flow theory—goal orientation theory: positive experience is related to athlete’s goal orientation. Front Psychol..

