# Supplementary material for: Getting into a “Flow” state: a systematic review of flow experience in neurological diseases
Source: J Neuroeng Rehabil. 2021 Apr 20;18:65. doi: 10.1186/s12984-021-00864-w (PMC8059246; doi:10.1186/s12984-021-00864-w)
Supplement: Supplementary file 1 — Additional file 1. Search strategy. [file 12984_2021_864_MOESM1_ESM.docx]

**Getting into a «Flow» state: a systematic review of flow experience in neurological diseases**

**Additional File 1: Search Strategy**

Date of searches: 30-06-2020

Databases:

Pubmed 165

Embase 33

Scopus 151

Cinhal: 558

Total: 907

Additional records identified through other scores: 4

Total: 911

**Pubmed:**

**#1 Stroke, traumatic brain injury, multiple sclerosis and Parkinson’s disease**

(“Neurological disease”[Mesh] OR neurorehabilitation[Mesh] “stroke” [tiab] OR “traumatic brain injury”[tiab] OR “multiple sclerosis”[tiab] OR “Parkinson”[tiab])

**#2 Flow experience**

(“positive psychology”[Mesh] OR “flow questionnaire”[Mesh] OR “flow exper*”[ti.ab] OR “flow theor*”(tiab) NOT (“cereb* flow”[tiab] OR dyn* flow[tiab] OR “exp* flow”[tiab] OR “blood flow”[tiab] OR “venous flow”[tiab])

**#3**

((flow exp*) NOT (cereb* flow OR dyn* flow OR exp* flow OR blood flow OR venous flow)) AND stroke

**#4**

((flow exp*) NOT (cereb* flow OR dyn* flow OR exp* flow OR blood flow OR venous flow)) AND traumatic brain injury

**#5**

((flow exp*) NOT (cereb* flow OR dyn* flow OR exp* flow OR blood flow OR venous flow)) AND multiple sclerosis

**#6**

((flow exp*) NOT (cereb* flow OR dyn* flow OR exp* flow OR blood flow OR venous flow)) AND parkinson

**Embase:**

**#1 Stroke, traumatic brain injury, multiple sclerosis and Parkinson’s disease**

‘Neurological disease’/exp OR ‘neurorehabilitation’/exp OR ‘stroke’:ti,ab OR ‘traumatic brain injury’:ti,ab OR ‘multiple sclerosis’:ti,ab OR ‘parkinson’:ti,ab

AND

**#2 Flow experience**

‘positive psychology’/exp OR ‘flow questionnaire’/exp OR ‘flow exper*’:ti,ab OR ‘flow theor*’:ti,ab NOT (‘cereb* flow’:ti,ab OR ‘dyn* flow’:ti,ab OR ‘exp* flow’:ti,ab OR ‘blood flow’:ti,ab OR ‘venous flow’:ti,ab)

**#3**

‘flow exp*’/exp NOT ‘cereb* flow’ OR ‘dyn* flow’ OR ‘exp* flow’ OR ‘blood flow’ OR ‘venous flow’ AND ‘stroke’:ti,ab

**#4**

‘flow exp*’/exp NOT ‘cereb* flow’ OR ‘dyn* flow’ OR ‘exp* flow’ OR ‘blood flow’ OR ‘venous flow’ AND ‘traumatic brain injury’:ti,ab

**#5**

‘flow exp*’/exp NOT ‘cereb* flow’ OR ‘dyn* flow’ OR ‘exp* flow’ OR ‘blood flow’ OR ‘venous flow’ AND ‘multiple sclerosis’:ti,ab

**#6**

‘flow exp*’/exp NOT ‘cereb* flow’ OR ‘dyn* flow’ OR ‘exp* flow’ OR ‘blood flow’ OR ‘venous flow’ AND ‘parkinson’:ti,ab

Scopus:

**#1 Stroke, traumatic brain injury, multiple sclerosis and Parkinson’s disease**

stroke OR traumatic brain injury OR multiple sclerosis OR Parkinson OR neurological disease*

AND

**#2 Flow experience**

Flow exper* OR flow questionnaire OR flow theor* NOT cereb* flow OR dyn* flow OR exp* flow OR blood flow OR venous flow

**#3**

‘flow exp*’/exp NOT ‘cereb* flow’ OR ‘dyn* flow’ OR ‘exp* flow’ OR ‘blood flow’ OR ‘venous flow’ AND ‘stroke’:ti,ab

**#4**

‘flow exp*’/exp NOT ‘cereb* flow’ OR ‘dyn* flow’ OR ‘exp* flow’ OR ‘blood flow’ OR ‘venous flow’ AND ‘traumatic brain injury’:ti,ab

**#5**

‘flow exp*’/exp NOT ‘cereb* flow’ OR ‘dyn* flow’ OR ‘exp* flow’ OR ‘blood flow’ OR ‘venous flow’ AND ‘multiple sclerosis’:ti,ab

**#6**

‘flow exp*’/exp NOT ‘cereb* flow’ OR ‘dyn* flow’ OR ‘exp* flow’ OR ‘blood flow’ OR ‘venous flow’ AND ‘parkinson’:ti,ab

Cinhal:

**#1 Stroke, traumatic brain injury, multiple sclerosis and Parkinson’s disease**

stroke OR traumatic brain injury OR multiple sclerosis OR Parkinson OR neurological disease*

AND

**#2 Flow experience**

Flow exper* OR flow questionnaire OR flow theor* NOT cereb* flow OR dyn* flow OR exp* flow OR blood flow OR venous flow

**#3**

‘flow exp*’/exp NOT ‘cereb* flow’ OR ‘dyn* flow’ OR ‘exp* flow’ OR ‘blood flow’ OR ‘venous flow’ AND ‘stroke’:ti,ab

**#4**

‘flow exp*’/exp NOT ‘cereb* flow’ OR ‘dyn* flow’ OR ‘exp* flow’ OR ‘blood flow’ OR ‘venous flow’ AND ‘traumatic brain injury’:ti,ab

**#5**

‘flow exp*’/exp NOT ‘cereb* flow’ OR ‘dyn* flow’ OR ‘exp* flow’ OR ‘blood flow’ OR ‘venous flow’ AND ‘multiple sclerosis’:ti,ab

**#6**

‘flow exp*’/exp NOT ‘cereb* flow’ OR ‘dyn* flow’ OR ‘exp* flow’ OR ‘blood flow’ OR ‘venous flow’ AND ‘parkinson’:ti,ab
