# Supplementary material for: Getting into a “Flow” state: a systematic review of flow experience in neurological diseases
Source: J Neuroeng Rehabil. 2021 Apr 20;18:65. doi: 10.1186/s12984-021-00864-w (PMC8059246; doi:10.1186/s12984-021-00864-w)
Supplement: Supplementary file 2 — Additional file 2. Methodology quality and results of flow questionnaires per measurement properties and the rating criteria for good measurement properties. [file 12984_2021_864_MOESM2_ESM.docx]

**Getting into a «Flow» state: a systematic review of flow experience in neurological diseases**

**Additional File 2 Methodology quality and results of flow questionnaires per measurement properties**

Methodology quality (Meth qual) of included studies per measurement properties is rated based on the COSMIN checklist with a four-point scale “very good” (VG), “adequate” (A), “doubtful” (D), “inadequate” (I) or “not applicable” (NA). Each result is rated based on the COSMIN checklist either sufficient (+), insufficient (-), or indeterminate (?). Empty boxes mean that no data are reported, or it was not analysed. The rating criteria for good measurement properties are presented below.

| **Additional File 2:** Rating criteria for good measurement properties following the COSMIN guidelines (Prinsen et al., 2018) | | |
| --- | --- | --- |
| **Measurement property** | **Rating^1^** | **Criteria** |
| **Construct validity** |  |  |
| Structural validity | + | CTT:  CFA: CFI or TLI or comparable measure > 0.95 OR RMSEA <0.06 OR SRMR <0.08^2^ |
|  | ? | CTT: Not all information for '+' reported |
|  | - | Criteria for '+' not met |
|  | + | The result is in accordance with the hypothesis |
| Hypotheses testing for construct validity | ? | No hypothesis defined (by the review team) |
|  | - | The result is not in accordance with the hypothesis |
|  | + | No important differences found between group factors (such as age, gender, language) in multiple group factor analysis OR no important DIF for group factors (McFadden's R^2^ <0.02) |
| Cross-Cultural validity | + | No multiple group factor analysis OR DIF analysis performed |
|  | - | Important differences between group factors OR DIF was found |
| **Reliability** |  |  |
|  | + | At least low evidence^4^ for sufficient structural validity^5^ AND Cronbach's alpha(s) > 0.70 for each unidimensional scale or subscale |
| Internal consistency | ? | Criteria for "At least low evidence^4^ for sufficient structural validity^5^" not met |
|  | - | At least low evidence4 for sufficient structural validity5 AND Cronbach's alpha(s) < 0.70 for each unidimensional scale or subscale |
|  | + | SDC or LoA < MIC^5^ |
| Measurement error | ? | MIC not defined |
|  | - | SDC or LoA > MIC^5^ |
|  | + | The result is in accordance with the hypothesis^7^ OR AUC > 0.70 |
| **Responsiveness** | ? | No hypothesis defined (by the review team) |
|  | - | The result is not in accordance with the hypothesis7 OR AUC > 0.70 |

AUC = are under the curve; CFA = Confirmatory Factor Analysis; CFI = Comparative Fit Index; CTT = Classical Test Theory; DIF = Differential Item Functioning; LoA = Limits of Agreement, MIC = Minimal Important Change; RMSEA = Root Mean Square Error of Approximation; SDC = Smallest Detectable Change; SRMR = Standardized Root Mean Square Residual; TLI = Tucker-Lewis Index

^1^ '+' = sufficient, '-' insufficient, '?' = indeterminate

^2^ To rate the quality of the summary score, the factor structures should be equal across studies

^4^ As defined by grading the evidence according to the GRADE approach

^5^ This evidence may come from different studies

^7^ The results of all studies should be taken together, and it should then be decided if 75% of the results are in accordance with the hypotheses

| **Content Validity** | | | | | | | | | | | | | |
| --- | --- | --- | --- | --- | --- | --- | --- | --- | --- | --- | --- | --- | --- |
|  | Meth  qual | Relevance | | | | | | Comprehensiveness | Comprehensibility | | | | |
|  |  | Results (rating) | | | | | | Results (rating) | Results (rating) | | | | |
|  |  | Are the in-  cluded items relevant for  the construct  of interest? | Are the in-  cluded items relevant for  the target population of interest? | Are the in-  cluded items  relevant for  the context of  use of interest? | Are the  response  options appropriate? | Is the  recall period appro-  priate? | Relevance overall rating | Are all key  concepts  included? | Are the PROM items understood by the population of  interest as intended? | Are the  PROM items and response options understood  by the population  of interest as intended? | Are the PROM  items appropria-  tely worded? | Do the  response  options  match the question? | Comprehensibility  overall rating |
| Flow in human computer interactions (Webster et al., 1993) | I | - | ? | ? | + |  | - | - |  |  | + | + | - |
| FSS (Jackson, 1992, 1995; Jackson & Marsh, 1996) | VG | + | + | + | + |  | + | + | + | + | + | + | + |
| FSS Greek (Doganis et al., 2000) | A | + | + | + | + |  | + | + |  |  |  | + | - |
| FSS Greek (Stavrou & Zervas, 2004) | VG | + | + | + | + |  | + | + | + | + | + | + | + |
| FSS Spanish (García Calvo et al., 2008) | A | + | + | + | + |  | + | + |  |  | + | + | - |
| SFS (Martin & Jackson, 2008) | A | + | + | + | + |  | + | + |  |  | + | + | - |
| CFS (Martin & Jackson, 2008) | A | + | + | + | + |  | + | + |  |  | + | + | - |
| FSSOT (Yoshida et al., 2013) | A | + | + | + | + |  | + | + |  |  | + | + | - |

| **Construct Validity** | | | | | | | | | | | | | | | | |
| --- | --- | --- | --- | --- | --- | --- | --- | --- | --- | --- | --- | --- | --- | --- | --- | --- |
|  |  | Structural validity | | | | | | | | | Hypotheses testing | | | Cross-Cultural validity | | |
|  |  | n | Meth  qual | Results (rating) | | | | | | | N | Meth  Qual | Results (rating) | N | Meth  qual | Results (rating) |
|  | Language |  |  |  | Model | X / df | CFI or RNI a) | NNFI or TLI b) | RMSEA | SRMR |  |  |  |  |  | MI or DIF |
| Flow in human-computer interactions  (Webster et al., 1993) | E | 133 | A | CFA | 4 Factors explaining 53.4% of  the variances | 108.97/2.3 |  |  | 0.09 (+) |  |  |  |  |  |  |  |
| FSS  (Jackson & Marsh, 1996) | E | 394 | VG | CFA | 1 First order  9 First order  1 Higher order +  9 First order | 3447.37/59  1124.95/585  1254.21/585 | .573 a)  (-)  .915 a)  (-)  .900 a)  (-) | .547  (-)  .904  (-)  .892  (-) | .112  (-)  .051  (+)  .055  (+) |  | 213 | VG | Perceived sport ability (r=-0.11-0.40) (-)  Total anxiety (r=-0.37 - -0.17) (-)  Anxiety-somatic (r=-0.24 - -0.14) (-)  Anxiety Concentration disruption (r=-0.49 - -0.08) (-)  Anxiety-worry (r=-0.36 - 0.18) (-)  Intrinsic motivation to experience stimulation (r=0.05 - 0.25) (-) |  |  |  |
| FSS  (Marsh & Jackson, 1999) | E | 385 | VG | CFA | 1 First order  9 First order  1 Higher order +  9 First order | 4835.44/560  1128.12/524  1262.60/551 | .447 a)  (-)  .922 a)  (-)  .908 a)  (-) | .413 b)  (-)  .911 b)  (-)  .901 b)  (-) | .141  (-)  .050  (+)  .054  (+) |  |  |  |  |  |  |  |
| FSS (Vlachopoulos et al., 2000) | E | 1231 | VG | CFA | 1 First order  9 First order  1 Higher order +  9 First order | 8346/594  2626/558  3044/585 | .636  (-)  .903  (-)  .885  (-) | .614  (-)  .890  (-)  .876  (-) | .103  (-)  .055  (+)  .058  (+) | .083  (-)  .051  (+)  .061  (+) |  |  |  |  |  |  |
| FSS (Doganis et al., 2000) | G | 134 | I | CFA | 9 Factor Model | 803.63/558 | .868  (-) |  | .063  (-) |  |  |  |  |  | I | Translation and  Back-Translation; Discussion on final wording (?) |
| continued | | | | | | | | | | | | | | | | |

| Construct Validity continued | | | | | | | | | | | | | | | | |
| --- | --- | --- | --- | --- | --- | --- | --- | --- | --- | --- | --- | --- | --- | --- | --- | --- |
| FSS (Stavrou & Zervas, 2004) | G | 385 | VG | CFA | 9 First order  1 Higher order +  9 First order | 1237.691/558  1530.687/576 | .901  (-)  .887  (-) | .888  (-)  .876  (-) | .059  (+)  .071  (+) | .063  (+)  .070  (+) | 385 | VG | Subjective Measure of Performance (r = 0.03 - 0.65; M = 0.35) less than 75% (-)  Trait Sport Anxiety (r = -0.34 – 0.19; M = -0.12) (-)  Trait-Sport Confidence (r = -0.08 – 0.40; M 0.26) (-)  Task-Orientation (r = -0.01 – 0.13; M 0.03) (-)  Ego-Orientation (r = 0.1 – 0.28; M = 0.19) (-)  State Cognitive Anxiety 1 day before competition (r = -0.48 – 0.18; M -0.16) (-)  State Cognitive Anxiety 1 hour before competition (r = -0.46 – 0.18; M -0.17) (-)  State Cognitive Anxiety during competition (r = -0.46 – 0.20; M -0.19) (-)  State Somatic Anxiety 1 day before competition (r = -0.19 – 0.28; M -0.05) (-)  State Somatic Anxiety 1 hour before competition (r = -0.22 – 0.27; M -0.06) (-)  State Somatic Anxiety during competition (r = -0.21 – 0.29; M -0.08) (-)  State Self Confidence 1 day before competition (r = -0.12 – 0.38; M 0.26) (-)  State Self Confidence 1 hour before competition (r = -0.09 – 0.48; M 0.31) (+)  State Self Confidence during competition (r = -0.08 – 0.58; M 0.38) (+) | 20 | A | Translation – Content analysis – structured open-ended interview, Logical validity with  20 athletes (?) |
| FSS (García Calvo et al., 2008) | S | 2036 | VG | CFA | Hierarchical model with 1 principal and 9 second order factors  9 First order | 4253.746/585  3763.006/558 | .91  (-)  .92  (-) | .90 b)  (-)  .91 b)  (-) | .054  (+)  .052  (+) | .051  (+)  .048  (+) |  |  |  |  | I | Translation and  Back-Translation, discussion in team in final wording of items  (?) |
| SFS  (Martin & Jackson, 2008) | E | 637  239  224 | VG | CFA | Independent 9 items (all parameters are free)  Work  Sport  Music | 136.78/27  112.38/27  45.11/27 | .94  (-)  .93  (-)  .99  (+) | .92  (-)  .91  (-)  .98  (+) | .08  (-)  .12  (-)  .06  (-) | .05  (+)  .06  (+)  .04  (+) |  |  | Participation (r .74 work, r .90 sport, r .80 music) (+)  Enjoyment (r 82 work, r .89 sport, r .73 music) (+)  Buoyancy (r .81 work, r .74 sport, r .68 music) (+)  Aspirations (r .71 work, r .81 sport, r .73 music) (+)  Adaptive cognition (r .72 work, r .73 sport, r .82 music) (+)  Adaptive behaviours (r .59 work, r .69 sport, r .70 music) (+)  Impede/maladapt behaviours (r -.59 work, r -.37 sport, r -.49 music) (+)  Maladaptive behaviours (r -.70 work, r -.47 sport, r -.60 music) (+) |  |  |  |
| continued | | | | | | | | | | | | | | | | |

| Construct Validity continued | | | | | | | | | | | | | | | | |
| --- | --- | --- | --- | --- | --- | --- | --- | --- | --- | --- | --- | --- | --- | --- | --- | --- |
| CFS  (Martin & Jackson, 2008) | E | 2229  378  220 | VG | CFA | Independent 9 items (all parameters are free)  General school  Mathematics  Extracurricular Activity  Sport | 585.8/35  172.73/35  590.18/35  124.79/35 | .98  (+)  .98  (+)  .98  (+)  .97  (+) | .98  (+)  .97  (+)  .97  (+)  .96  (+) | .08  (-)  .10  (-)  .08  (-)  .11  (-) | .04  (+)  .04  (+)  .03  (+)  .05  (+) |  |  | Participation (r .56 general school, r .49 mathematics, r .25 extracurricular) (+)  Enjoyment (r .71 general school, r .58 mathematics, r .13 extracurricular) (+ (except extracurricular))  Buoyancy (r .42 general school); aspirations (r .68 general school, r .42 mathematics) (+)  Adaptive cognition (r .74 general school, r .67 mathematics) (+)  Adaptive behaviours (r .83 general school, r .68 mathematics) (+)  Buoyancy (r .15 mathematics; r .20 extracurricular) (-)  Aspiration (r .12 extracurricular), Adaptive cognition (r .23 extracurricular)  Adaptive behaviours (r .18 extracurricular)  Maladaptive behaviours (r -.79 general school, r -.72 mathematics)  Impede/maladapt behaviours (r -.11 general school, r -.23 mathematics, r -.10 extracurricular)  Maladaptive behaviours (r -.15 extracurricular) |  |  |  |
| FSSOT (Yoshida et al., 2013) | J | 240 | VG | EFA  CFA | 3 Factor explaining 58.62% of the variances  3 Factor Model  GFI .89  NFI .904 | 200.06/74 | .937  (-) | .922  (-) | .084  (-) |  | 204 | VG | Anxiety total r .537 (+)  Anxiety absent r -.611 (+)  Anxiety present r -.088 (-) |  |  |  |

| **Reliability** | | | | | | | **Responsiveness** | | |
| --- | --- | --- | --- | --- | --- | --- | --- | --- | --- |
|  | Internal consistency | | | Measurement Error | | |  |  |  |
|  | n | Meth  qual | Results (rating) | N | Meth  Qual | Results (rating) | N | Meth  qual | Results (rating) |
| Flow in human-computer interactions  (Webster et al., 1993) | 133 | A | 0.82 (+) |  |  |  |  |  |  |
| FSS  (Jackson & Marsh, 1996) | 394 | VG | .80 - .86 (+)  M = .83 (+) |  |  |  |  |  |  |
| FSS (Marsh & Jackson, 1999) |  |  |  |  |  |  |  |  |  |
| FSS (Vlachopoulos et al., 2000) | 1231 | VG | .78 - .84 (+) except time factor .65 (-) |  |  |  |  |  |  |
| FSS Greek (Doganis et al., 2000) | 134 | VG | .34 - .88 (-) |  |  |  |  |  |  |
| FSS Greek (Stavrou & Zervas, 2004) | 385 | VG | .75 - .92 (+)  Mean .82 (+) |  |  |  |  |  |  |
| FSS Spanish (García Calvo et al., 2008) | 2036 | A | Over .70 (data not presented) (?) |  |  |  |  |  |  |
| SFS (Martin & Jackson, 2008) | 637  239  224 | VG | Work .73 (+)  Sport .83 (+)  Music .84 (+) |  |  |  |  |  |  |
| CFS (Martin & Jackson, 2008) | 2229  378  220 | VG | General School .93 (+)  Mathematics.94 (+)  Extracurricular Activity .91 (+)  Sport .92 (+) |  |  |  |  |  |  |
| FSSOT (Yoshida et al., 2013) | 240 | VG | .867 - .871 (+) |  |  |  |  |  |  |

CFA = Confirmatory Factor Analysis; CFI = Comparative Fit Index; CFS = Core Flow Scale; DIF = Differential Item Functioning; df = degree of freedom; E = English; EFA = Exploratory Factor Analysis; FSS = Flow State Scale; FSSOT = Flow State Scale for Occupational Tasks; G = Greek; IFI = Incremental Fit Index; J = Japanese; MI = Measurement Invariance; NA = not applicable; NNFI = Non-normed Fit Index; PROM = Patient‐Reported Outcome Measure; RCFI = Robust Comparative Fit Index; RNI = Relative Noncentrality Index; RMSEA = Root Mean Square Error of Approximation; S = Spanish; SFS = Short Flow Scale; SRMR = Standardized Root Mean Square Residual; r = correlation coefficient; X2 = Chi square
